# Ocular conditions and injuries, detection and management in spaceflight

**DOI:** 10.1038/s41526-023-00279-y

**Published:** 2023-05-16

**Authors:** Elana Meer, Seanna Grob, Erik L. Antonsen, Aenor Sawyer

**Affiliations:** 1 Department of Ophthalmology, University of California San Francisco, San Francisco, CA USA; 2 University of California Space Health Program, San Francisco, CA USA; 3 Department of Emergency Medicine and Center for Space Medicine, Baylor College of Medicine, Houstan, Texas USA; 4 Department of Orthopaedic Surgery, University of California San Francisco, San Francisco, CA USA

**Keywords:** Conjunctival diseases, Eye abnormalities, Trauma, Corneal diseases, Eyelid diseases

## Abstract

Ocular trauma or other ocular conditions can be significantly debilitating in space. A literature review of over 100 articles and NASA evidence books, queried for eye related trauma, conditions, and exposures was conducted. Ocular trauma and conditions during NASA space missions during the Space Shuttle Program and ISS through Expedition 13 in 2006 were reviewed. There were 70 corneal abrasions, 4 dry eyes, 4 eye debris, 5 complaints of ocular irritation, 6 chemical burns, and 5 ocular infections noted. Unique exposures on spaceflight, such as foreign bodies, including celestial dust, which may infiltrate the habitat and contact the ocular surface, as well as chemical and thermal injuries due to prolonged CO2 and heat exposure were reported. Diagnostic modalities used to evaluate the above conditions in space flight include vision questionnaires, visual acuity and Amsler grid testing, fundoscopy, orbital ultrasound, and ocular coherence tomography. Several types of ocular injuries and conditions, mostly affecting the anterior segment, are reported. Further research is necessary to understand the greatest ocular risks that astronauts face and how better we can prevent, but also diagnose and treat these conditions in space.

## Introduction

The NASA Critical Path Roadmap Project determined trauma and acute medical illness events to be at the highest level of risk in terms of predicted incidence versus potential impact on mission and crew health^1^. Since that time, acute and traumatic medical conditions have been bookkept by NASA in the In-Flight Medical Conditions risk and adjudicated by the Human System Risk Board at NASA Johnson Space Center^2–4^. Although the Roadmap Project historically determined that trauma was the highest level of risk, ocular trauma in spaceflight has not been a primary driving factor for medical to date. However, future missions are likely to have different characteristics from missions occurring in the last 60 years. There are currently three companies funded to build space stations in LEO, and NASA and private companies are currently working towards a long term return to the moon and missions to Mars. The types of tasking for astronauts are important when considering ocular risk. Increased construction-type work for orbital and lunar habitats are expected to bring different condition incidences for many medical concerns. The consequences of ocular injury for current mission critical tasks like orbital piloting/maneuvering, EVA functionality for construction and maintenance, and scientific tasks that are likely to become larger parts of future astronaut work requirements are highly dependent on ocular health.

This in-flight medical risk serves as an organizing point for Crew Health and Performance (CHP) System trades across the broad variety of potential medical issues that can arise in a human spaceflight mission. Space Flight-Associated Neuro-Ocular Syndrome (SANS) is frequently studied and discussed as a cause of vision changes in astronauts. Separate from SANS, ocular trauma and many other ocular conditions can cause irritation or visual compromise for astronauts, including bacterial corneal ulcers, corneal abrasions, and corneal foreign bodies^5,6^. All of these risks are currently considered ‘Red’ or high risk for an eventual Mars mission and the In-flight Medical Risk is held at ‘Red’ for long duration lunar missions as well^2,3^. The NASA evidence books have outlined the importance of considering the many potential sources of ocular trauma and irritation from celestial dust exposure to chemical injury. However, the diagnostic and treatment modalities to evaluate and treat the anterior segment of the eye are limited on ISS. This is a consequence of the mass and volume limitations that are associated with any human spaceflight mission and prioritization of needs based on perceived risk. The decision on what to include in the CHP systems of future missions becomes more challenging as those mass and volume allocations decrease while exposure to the spaceflight environment increases^7^. Making risk-informed decisions about what to include in the CHP system for future missions depends on careful assessment of the historical evidence that can inform our understanding of the likelihood and consequence of ocular health issues in spaceflight^8,9^.

In this project, we aim to characterize the potential ocular injuries in space, review the literature surrounding them, and discuss the current diagnostic and treatment modalities currently available and for future consideration in space flight. By providing a review of the ocular injury, detection, and management in space flight, as well as future directions based on current innovations in the field of ophthalmology, we hope to encourage a holistic team approach between NASA and the ophthalmology community to improve ocular health in space flight.

## Review

### Systematic review and methodology

First, a literature review was conducted. Two databases, Pubmed and Google Scholar, were searched using the using the following search terms ((ocular injuries) OR ((trauma) AND (eye))) AND ((spaceflight) OR (astronaut) OR (microgravity)) OR (technology) OR (novel technology) OR (monitoring) AND (ENGLISH[Language]). Results were imported into Mendeley citation manager. Literature was excluded at the abstract stage on the grounds of: non-human studies, text not available in English, conference abstract only. Literature was included on the grounds of relevance to the given topic including search terms such as ocular injury in space, ultrasound assessment in space, corneal abrasion in space, corneal abrasion, dry eye in space, etc. This included methods for monitoring of conditions related to ocular complaints in space, and any published studies from populations considered to be analogs. We focused on ocular conditions other than SANS, which has been extensively reviewed in prior studies. Therefore SANS related literature was also excluded to focus on lesser known/reviewed ocular conditions affecting astronauts in space flight.

This study also included a comprehensive review of the NASA evidence books entitled “Artificial Gravity Countermeasure Evidence Reports,” “CNS, behavioral Health and Sensorimotor CBS Integrated Research Plan: Implementation Strategy and Problem Statement,” “Risk of Adverse Health and Performance Effects of Celestial Dust Exposure,” “Risk of Adverse Health Outcomes and Decrements in Performance due to Inflight Medical Conditions” “Risk of Spaceflight Associated Neuro-Ocular Syndrome.” Each evidence book was read and queried for eye related symptomatology, conditions, and exposures.

The following sections discuss first the ocular injuries and conditions that can occur in space with some discussion of spaceflight relevance and terrestrial treatment expectations. This is followed by a section dedicated to diagnostic modalities that are potential candidates for CHP systems in future human spaceflight. Potential value and limitations of these are considered in the context of spaceflight constraints. Finally therapeutic modalities are discussed including relevant treatments and preventive strategies.

As this was a review of publically available evidence reports, and review of published manuscripts, no IRB or NASA review was required.

### Characterization of ocular injuries and conditions in space

The Exploration Medical Conditions List at NASA has been the source of medical conditions considered for probabilistic risk analysis regarding medical risk in the past^10^. This list has historically included four eye specific conditions: Eye Chemical Burn, Eye Corneal Ulcer, Eye Infection, and Retinal Detachment. In one assessment, Eye Chemical Burn was the top influential medical condition contributing to the probability of evacuation for certain ISS mission conditions^10^. Exemplified by the medical condition and symptomatology reported from NASA evidence books (Table 1), it is important to expand consideration of ocular conditions beyond those traditionally considered for LEO missions to appropriately identify and mitigate risk in this domain. As commercial missions in Low Earth Orbit (LEO) begin and NASA missions beyond LEO are planned, this investigation and review of ocular conditions and injuries in spaceflight becomes even more important.

**Table 1:** Medical condition and symptomatology during NASA Space Missions Obtained from Lifetime Surveillance of Astronaut Health (LSAH) records for medical conditions that occurred among US astronauts during the Space Shuttle Program and ISS through Expedition 13 in 2006.

Medical Condition/Symptoms	Number of Events
Eye Abnormality unspecified	9
Eye Debris	4
Dry Eyes	4
Eye Irritation	5
Puffy eyes	1
Eye Abrasion (Foreign Body)	70
Eye Chemical Burn	6
Eye Infection	5

Data derived extracted from NASA Evidence books^4^.

### Corneal abrasions and foreign bodies

#### Definition of injury

Crew members on ISS or future lunar and planetary missions may be exposed to particles, in particular celestial dust (on Artemis and Mars missions), in a variety of ways that can be a risk for ocular foreign body or corneal abrasion^11^. Corneal abrasions are characterized as superficial corneal defects, classically caused by foreign bodies in the eye or minor blunt trauma. Of note, conjunctival foreign bodies are also possible and have similar symptomatology. These could be embedded in the conjunctiva or cause a conjunctival laceration. Foreign bodies may also penetrate the sclera causing a partial thickness or full thickness injury, also known as an open globe (see “Globe disruption/orbit penetration”).

#### Injury and exposures in spaceflight

Crew members may be exposed to the dust through extravehicular activities (EVAs), defined as any activity done by an astronaut or cosmonaut outside a spacecraft beyond the Earth’s appreciable atmosphere such as a spacewalk^12^. This can be secondary to the planetary surface work itself during which suit breakdown or any debris trapped in the suit could cause persistent irritation without ability to manually rub the eye or try to protect from debris exposure and to the return to the ship or while unpacking cargo. An EVA may be undertaken by crew members to install new equipment, or carry out repairs, maintenance, or fault investigation. When a crew leaves the surface of a celestial body and returns to microgravity, the dust can be introduced into the spacecraft as it can collect on spacesuits and boots. This dust will then float on return to the vehicle, which increases the opportunity for ocular exposure and subsequent injury^13–16^. Cleaning of suits in between the EVAs and changing of the environmental control life support system filters may also introduce celestial dust into their environment. Of note, foreign bodies trapped in the suit may be different from celestial dust. Spacesuits themselves may abrade skin, providing another source of exposure as celestial dust can penetrate through areas of epithelial erosion and abrasion. Celestial dust can also gain access to suits interior, as was the case during the Apollo missions, which is an additional source of dust exposure^4^.

Celestial dust exposure, in particular lunar dust, is important since the most commonly reported eye injury in space was eye abrasion secondary to foreign body (Table 1)^11^. Lunar dust also has particular properties predisposing it to causing irritation or abrasion as it has sharp edges. Mars dust has perchlorates which further irritate the eye. Similarly corneal irritation is considered a common/anticipated condition in space flight based on three-crew 13-month return journeys with 1 month on surface of another planet. Studies investigating lunar dust simulants and authentic lunar dust have aimed to determine the unique properties that may cause ocular irritation and to establish a permissible exposure limit for trips to the lunar surface of <6 months^11^. Authentic lunar soils from highland (Apollo 16) and mare (Apollo 17) regions have demonstrated lunar soil to be particularly abrasive to skin, more toxic than titanium dioxide^11,17,18^. During the Apollo mission, crews reported irritation of skin and eyes, however, data outlining the dermal and ocular hazards of lunar dust is limited. Furthermore, not all instances of irritation aligned with abrasion and visa versa, which suggests a spectrum of effects from conjunctival irritation to corneal abrasion^11^.

However, there is a disconnect between the symptomatology of ocular irritation and the demonstration of ocular toxicity in studies. Studies have applied respirable sized, jet-milled dust maintained in dry nitrogen until use, aimed to mimic the lunar dust toxicity, to the surface of cultured human keratinocytes. However, only minimal irritancy was demonstrated by this assay for lunar dust as measured by cell viability, suggesting that no special precautions would be recommended to protect against ocular exposures^15^. Further testing in vivo was performed in rabbits to assess acute irritation in the intact eye by pouring 0.1 mL volume of lunar dust into the conjunctival sac^15^. No corneal abrasions were observed by fluorescein staining, no adverse signs or symptoms were noted on the cornea, iris, or conjunctiva at any of the subsequent observation times (24, 48, and 72 h) with only minimal irritation (slight redness and swelling of the conjunctiva at 1 h after exposure without any adverse effects on the cornea, iris, or conjunctiva at 24, 48, and 72 h)^15^. As in past studies, symptoms were limited to minor acute effects of redness, itching, foreign body sensation, and discharge, which were treated with eye drops^19^. This lack of irritation was thought to be secondary to lunar grains becoming smaller, and more rounded in shape decreasing the level of abrasiveness. However, it is difficult to mimic the flow of lunar dust into the eye in Earth gravity settings. For example, depending on the mechanism of dust exposure, there is a variety of different speeds and entries of dust particles onto the ocular surface apart from pooling into the conjunctiva. Additionally, astronauts are likely exposed to the dust for longer intervals and for a more extended period of time than demonstrated in these studies. Therefore, these studies may not be able to accurately capture the abrasiveness of the particles onto the eye and the effects of long-term exposure.

Apart from mechanical irritation, it is also possible that the cornea may be adversely affected by molecular changes in the setting of chronic exposure to low levels of dust with surface features facilitating oxidative damage. Recent work has shown that even chronic insult of lunar dust as low as 20 mg/m^3^ elicits a molecular response in cornea tissue^20^. Therefore, while the macroscopic signs of mechanical irritancy and cytotoxicity may be limited, lunar dust exposure may affect a multitude of pathways in ocular tissue including oxidative stress response, mitochondrial dysfunction, fibrosis, epithelial healing, TGF-beta signaling, extracellular matrix remodeling, cellular proliferation, and eye development^20^. Compounding these molecular effects, lunar dust on the surface of the moon has been found to have added properties of ionization and activation, potentially exacerbating further damage to the cornea and increasing sensitivity to environmental insults^20^.

With future exploration missions to the moon and mars on the horizon, considerations of such dust exposure are crucial. Planetary surfaces are covered by a hard, abrasive dust and loose rock known as regolith, which have been extensively studied for their composition^15,21–25^. While studies have focused on artificial lunar dust, more research is necessary to better understand the effects of lunar or Martian geology^17,18^. In addition, there may be unique health challenges associated with asteroid or martial dust exposures, including the effects of environmental factors such as windstorms, which may increase the likelihood of ocular irritation or injury^26,27^.

In addition, while lunar dust is of particularly high importance as NASA considers return trips to Mars, it is important to consider the multitude of other potential sources of ocular foreign body on spaceflight. Any suspended particles may enter crewmembers’ eyes, and the risk of injury from a foreign body is considered to be higher in microgravity because the particulates are suspended within the cabin and follow existing air currents^28^. Corneal abrasions reported in spaceflight have also occurred secondary to objects such as elastic cords snapping out of place^28^.

It is also important to note that while the above focuses on corneal abrasions/corneal disruption, the same risk factors apply to foreign bodies of the conjunctiva or sclera.

#### Diagnosis and treatment

Small abrasions or conjunctival foreign bodies are typically diagnosed via slit lamp examination with fluorescein dye. In terrestrial medicine, conjunctival foreign bodies may be removed by saline irrigation or with the use of a sterile needle, forceps, or golf club foreign body spud along with topical anesthesia and a slit lamp. However, removal of foreign bodies can be particularly difficult in the case of small dust particulates, which can be difficult to visualize without a microscopic view through the slit lamp^29^. If there is concern for globe disruption with a scleral or corneal foreign body, surgical intervention may be indicated (see “Globe disruption/orbit penetration”).

### Corneal infection and corneal ulcer

#### Definition of injury

Corneal abrasions or ocular foreign bodies may progress from keratitis, corneal inflammation, to corneal ulcer with or without infiltrate. A corneal ulcer is characterized as a defect of the corneal epithelium involving the underlying stroma. Corneal ulcers involve inflammation of the cornea, and are frequently associated with an inflammatory infiltrate as bacteria are able to enter through the corneal epithelial defect. Such pathology is considered to be a potentially vision threatening ocular emergency^30^.

#### Potential causes of injury and known data on injury in spaceflight

Risk factors for corneal infection and corneal ulcers include contact lens wear, trauma (including foreign bodies, chemical, and thermal injuries), contaminated ocular solutions, changes in the corneal surface such as from dry eye, altered ocular defense mechanisms (from topical and systemic immune suppression), corneal edema, and systemic factors including vitamin A and B12 insufficiency^31^.

Many of these risk factors are present on space flight. As discussed above, small foreign bodies, including celestial dust, may infiltrate the habitat and come in contact with the ocular surface. Chemical and thermal injuries may also occur in the setting of prolonged C02 and heat exposure. Both short- and long-duration space flight has been associated with immune system dysregulation^32^. In addition, microgravity-induced cephalad fluid shifts in humans, and molecular responses in mouse eyes following spaceflight effects have demonstrated a predisposition to corneal edema after long-duration space flight^33^. Spaceflight has also been shown to affect microbial growth and virulence, making prevention, early diagnosis and treatment of any ocular infection of even more importance. Therefore, even though data is limited on the true number of events of corneal infection and ulcer present on space flight, it is important to consider the diagnosis, treatment, and symptom management as infections and ulcers can include rapid onset of ocular pain, redness, photophobia, discharge and decreased vision affecting astronaut operations.

#### Diagnosis and treatment

Clinical diagnosis is classically made based off of clinical history and slit lamp examination showing the presence of corneal infiltrate with or without an anterior chamber reaction (cells, flare, fibrin, hypopyon). General treatment requires broad spectrum topical antibiotic drops every 2–6 h, or at times fortified antibiotic drops hourly depending on the severity and location of the infection. Not only is this a challenging therapeutic regimen on ISS, but it is also possible to that the antibiotics themselves disrupt the surface of the corneal epithelium and increase risk of corneal surface irritation, and infection^31^. Furthermore, increasing antibiotic resistance may lead to ineffective antibacterial regimens.

### Lens dislocation

#### Definition of injury

A lens is defined as dislocated when it lies external to the hyaloid fossa, such as in the vitreous, in the anterior chamber, or on the retina. Lens subluxation refers to partial displacement of the lens, but the lens remains within the lens space. While dislocation of the natural lens is rare and tends to occur in the setting of certain systemic diseases, a direct blow to the eye could cause lens dislocation with trauma being the most common cause^34,35^. There are many important sight-threatening complications of lens subluxation such as increased intraocular pressure, in conditions of eyeball “flattening” during space flight. Complete dislocation of the lens into the vitreous body may lead to retinal edema, tear, or detachment with a concomitant significant drop in visual acuity.

#### Potential causes of injury and known data on injury in spaceflight

It is unclear to what extent space flight may predispose to lens dislocation or subluxation. The spectrum of ocular changes, including disc edema, globe flattening, choroidal folds, and hyperopic shift in refraction may all contribute to increased risk of lens dislocation or subluxation^36^. Studies have also shown the molecular effects of space flight on metabolic function of ocular tissue^37–41^, which could in turn affect laxity of the suspensory ligaments of the lens.

While to the best of our knowledge there had been no overt mention of lens dislocation on space flight, there have been studies during long duration space flight following astronauts who had previously undergone cataract surgery with placement of an intraocular lens implant. One astronaut developed a unilateral cataract unrelated to spaceflight and underwent phacoemulsification with insertion of an acrylic IOL on earth. Fifteen months later, he completed a 6-month mission without any documented change in IOL position during his space mission and excellent and stable vision during lift off^42^. While this is reassuring, conclusions drawn beyond that one astronaut are limited.

#### Diagnosis and treatment

Typically the ocular exam consists of visual acuity testing, external ocular exam, slit lamp exam, retinoscopy, refraction, and dilated corneas. In situations in which slit lamp exam is not feasible, penlight exam has been demonstrated to show iridodenesis, tremulousness of the iris, however, gold standard diagnosis does involve visualizing lens dislocation on slit lamp or on ocular ultrasound^43^. Since lens dislocation can progress to lens-induced glaucoma (with increased intraocular pressure), lens-induced inflammation or uveitis, inadequate visual acuity, and retinal detachment, prompt diagnosis and management is crucial^34^. Diagnosis involves checking visual acuity and complete ophthalmic examination including anterior segment and dilated fundus examination. If minimal displacement and vision not significantly affected, treatment may include observation. However, if significant dislocation, vision decline, or other sequelae, surgical intervention may be necessary^44^. In the setting of lens dislocation induced glaucoma, or increased intraocular pressure, medical management may include IOP lowering eye drops such as beta blockers (e.g. timolol), carbonic anhydrase inhibitors (e.g. dorzolamide), alpha agonist derivatives (e.g. brimonidine), and prostaglandin analogs (e.g. latanoprost), although there is limited data on efficacy in space flight.

### Globe disruption/orbit penetration

#### Definition of injury

Open globe injuries may occur secondary to blunt trauma when an object with sufficient momentum creates energy transfer over a larger surface area and increases the intraocular pressure inside the eye, causing an inside-out injury as the eye wall breaks down at its weakest point^45–48^. Open globe injuries may also occur secondary to lacerations, defined as a full thickness cut to the eye, either from a sharp object or foreign body, such as in penetrating or perforating injuries.

#### Potential causes of injury and known data on injury in spaceflight

While there has been no overt mention of open globe injuries in space, or penetration of ocular tissues in evidence-based reports, there are likely a number of scenarios where astronauts could suffer severe eye trauma while in space. For example, larger fragments coming off of cargo or particles approaching the eye at a faster speed on return of the vehicle could feasibly penetrate the globe or cause ocular injury. Concern for this mechanism of injury is further supported by the 70 reported events of foreign body in the eye (Table 1). Especially with longer and farther missions in the future, a plan should be in place for addressing such injuries.

#### Diagnosis and treatment

Diagnosis and treatment of open globe injuries would be especially complicated on the ISS. Typically in the setting of ocular trauma, a complete ophthalmic examination is necessary including visual acuity, confrontational visual fields, extraocular motility evaluation, color vision testing, and a detailed external, anterior segment and dilated fundoscopic examination. If there is significant concern for an open globe injury, intraocular pressure measurements may be avoided to prevent pressure on the globe. A penlight examination may assist in diagnosis and can provide clues to serious ocular injury, such as a peaked pupil. However, smaller wounds may necessitate a slit lamp examination with fluorescein testing to diagnose a full thickness corneal laceration or foreign body in the anterior chamber and more posterior wounds may require a dilated examination to visualize the wound or hemorrhage in the posterior segment of the eye. Ocular imaging, such as CT scan, is also important to rule out an intraocular foreign body and often will also show distortion of the natural round globe contour^49,50^. Ultrasonography is not usually recommended due to the concern for iatrogenic expulsion of globe contents^51^. Once a diagnosis is made, immediate surgery is indicated as any delays in surgical care may increase risk of endophthalmitis and/or choroidal hemorrhage^52^. Due to the diagnostic and treatment limitations on ISS, this would be challenging.

### Retinal/choroidal detachment

#### Definition

Retinal detachment is a vision-threatening condition in which subretinal fluid accumulates between the neurosensory and pigment epithelial layers of the retina. This may occur due to a break in the retina allowing for vitreous flow into the subretinal space, due to a proliferation on the surface of the retina or vitreous which causes traction pulling the neurosensory retina off of the retinal pigment epithelium, and/or due to inflammatory or exudative subretinal fluid collecting secondary to an adjacent inflammatory or mass lesion.

#### Potential causes of injury and known data on injury in spaceflight

Space flight has well characterized profound affects on the retina and choroid, as well as the associated vasculature. On a macroscopic level, choroidal folds, cephalad fluid shifts and edema, and globe flattening have been described in the literature, as well as a multitude of microscopic effects due to a combination of microgravity and environmental stressors^36^. Space flight elicits gene expression and histologic changes by inducing oxidative and cellular stress responses in the mouse retina affecting the expression of multiple genes responsible for triggering a protective response^33,37,53–56^. While the oxidative stress induced DNA damage and abnormalities in oxidative and cellular stress, cell death, and hypoxia have been demonstrated to be reversible on return to earth^53^, these effects may increase susceptibility to retinal or choroidal injury on ISS. Furthermore, studies have shown that total retinal, retinal pigment epithelium, and choroid layer thickness were significantly lower after space flight, suggesting that retinal performance may decrease over extended periods of spaceflight and cause visual impairment^56^. Exacerbating this predisposition are the many pressure stressors astronauts undergo in the setting of microgravity, pressurized space suits, and high resistance exercise^57^. Persons who are older, have high myopia, history of eye trauma or prior eye surgery, or family history of retinal detachment are also at higher risk for retinal tear in general, unrelated to spaceflight.

#### Diagnosis and treatment

Retinal examination to evaluate for retinal tears or other retinal pathology requires a complete dilated fundoscopic examination with scleral depression to visualize peripherally to the ora serrata. On ISS this may be challenging given the lack of equipment and ophthalmologic training. However, the potential for progression to blindness, vastly impacting astronauts ability to perform makes this an essential ophthalmologic condition to consider. Furthermore, treatment necessitates prompt surgical evaluation and management with techniques including pneumatic retinopexy, victrectomy, scleral buckles, and/or laser therapy.

### Occlusion of the retinal artery or central retinal vein

#### Definition

Retinal artery occlusions and central retinal vein occlusions are characterized by the blockage of blood to the retina of the eye causing a sudden loss of eyesight in that eye. While not classically an “injury,” it is important to discuss as certain key components of the space flight environment may increase the risk of this sight threatening condition.

#### Potential causes of injury and known data on injury in spaceflight

As described, there are several metabolic changes underlying changes in the vasculature of the retina^58^. These changes in vessel caliber and density may be compounded by hypercoagulability in space^59–61^, increasing the risk for occlusion of the retina, an ophthalmologic emergency. Such occlusion of the retinal vascular lumen could occur by embolus, thrombus, or inflammatory/traumatic vessel wall damage or spasm and may lead to acute ischemia of the inner retina leading to atrophy and visual loss. The first case of deep venous thrombosis recorded in human spaceflight was published recently and found incidentally during a research study^62^. The research study in which it was identified also identified another possible thrombus in a separate astronaut. These findings have taken concerns about hypercoagulability in spaceflight from the theoretical into the practical domain.

#### Diagnosis and treatment

Diagnosis requires a dilated fundoscopic examination showing retinal whitening in the area of vascular occlusion, which may also include a cherry red spot in the setting of a central retinal artery occlusion or significant macular ischemia. Optical coherence tomography may reveal hyperreflectivity of the inner retina and fluorescein angiography may show a delay or absence of filling of the affected retinal arteries. Ocular massage, anterior chamber paracentesis, and oxygen to increase vasodilation and perfusion have been discussed and studied as treatment for retinal arterial occlusion^63^, but there is not enough evidence to confirm if any intervention is beneficial.

### Ocular chemical burn

#### Definition

Ocular chemical burns may occur secondary to a corrosive substance being introduced into the eye or periocular tissue.

#### Potential causes of injury and known data on injury in spaceflight

Prolonged Co2 exposure in space flight may lead to ocular toxicity^64^. This is exacerbated by the reduced air convection in microgravity leading to local pockets of C02 developing around the nose and mouth and facial area^57^. In probabilistic analysis of medical risks, eye chemical burn and burns secondary to fire are the most likely environmental causes of medical illness leading to evacuation on ISS to earth^10^. Ocular surface burns can range in severity from to corneal epithelial damage, corneal haze, total epithelial loss, to opacification of the cornea.

Chemical injuries may occur as a result of acid, alkali, or neutral agents. In particular acidic substances include sulfuric acid (found in car batteries), sulfurous acid (found in bleach and refrigerant), hydrofluoric acid (found in glass polishing and mineral refining), acetic acid (found in vinegar), and hydrochloric acid (found in swimming pools). Common alkali agents include ammonia (found in cleaning agents, fertilizers, and refrigerants), potassium hydroxide (found in caustic potash), lye (found in drain cleaners and airbags), magnesium hydroxide (found in firework sparklers, flares), and Lime (found in plaster, mortar, cement, white wash)^65,66^. These chemicals can also be found on spaceflight, as components of reagents used in a variety of research project from human research to biology and biotechnology to physical and materials sciences to technology development. In particular, there have been instances of accidental toxic potassium hydroxide exposure from the oxygen vent as well as nitrogen dioxide^67^. In addition, propulsion propellant (Freon, hydrazines, nitrogen dioxide) leaks into the spacecraft interior have been demonstrated to be toxic in even small quantities^68^. While this may sound hypothetical, combustion products and propellant including hydrazine and nitrogen oxide, and nitrogen tetroxide were sucked into the cabin on re-entry during the Apollo-Soyuz Test Project in July 1975^69^. These particular chemicals may also enter the spacecraft through the air lock or through crystallization on EVA suits^67^.

Heat degradation of electronic devices has also been shown to cause additional exposure on space flight. In particular, thermodegradation of spacegraft polymers produces formaldehyde and ammonia, with 9 incidents recorded from STS-35 to STS-55 and four resulting from electrical wiring^67^. Furthermore, follow up analysis found benzene, acetaldehyde, and dimethyl sulfides in the space shuttle crew compartment atmosphere^70^. Freon in cooling loops similarly poses a significant hazard, and while most pollutants reach a state of equilibrium within the first three to 4 days of a mission, hydrogen, methane, dichloromethane, and formaldehyde may not, all with potential systemic and ocular toxic effects^67^.

Thermal burns in the eye may result when the choroid layer blood flow is no longer able to appropriately regulate the temperature distribution of the retina. This may occur in the setting of chemical injuries, as well as ocular laser damage, leading to vascular damage and hemorrhage into the vitreous of the eye, leading to blurry vision and increased risk of retinal detachment.

#### Diagnosis and treatment

Copious irrigation is a crucial component of initial management in chemical burns, along with identifying the presence of and removing any caustic foreign bodies through inspection. The pH must be evaluated regularly and irrigation continued until the pH is brought back to 7. Volume of irrigation depends on the chemical insult and can range from 1–10 L required to regulate the pH. Providing adequate irrigation solution can be particularly challenging in spaceflight due to mass and volume limitations.

### Radiation injury

#### Definition

Radiation has been demonstrated to cause damage to parts of the eye leading to blurry vision, dry eyes, cataracts, retinal detachments, glaucoma, madarosis, abnormal vascularity, and optic neuropathy.

#### Potential causes of injury and known data on injury in spaceflight

Plans for human spaceflight to a near-Earth asteroid or journeys to Mars have brought concerns about the long-term health effects of space radiation to the forefront^71,72^. The space radiation environment in low Earth orbit, where the ISS is located, exposes astronauts to higher levels of radiation than that of the surface of earth. In low Earth orbit, the Earth’s magnetosphere provides substantial protection^57^. Exploration class missions beyond LEO lose the protection of Earth’s magnetosphere,exposing astronauts to greater levels galactic cosmic radiation^72^. The sequelae of such radiation exposure are broad with systemic effects potentially contributing to ocular injury. Exceedingly high levels of radiation exposure (20 GY) have been demonstrated to result in brain edema and neuroinflammation secondary to impaired brain-blood barrier function^73,74^, elevated intracranial pressure^75^. On mini-pig models, exposure to 2.5 Gy electron simulated solar particle events demonstrated higher CSF opening pressures 90-days after exposure^76^, and Sprague-Dawley rat models demonstrated greater oxidative stress in the retina and aortic vasculature after 3 Gy spread over 16 days with high dietary iron^77^. While these effects are thought to be proportional to the level of radiation exposure (higher Gy associated with greater oxidative stress on the retina), it is unclear if chronic low-dose low-linear energy transfer (LET) radiation from training may alter the blood brain or retinal barriers. Furthermore, it is also unclear whether the main component of galactic cosmic radiation, high-LET radiation, impacts ocular tissues differently than the low-LET radiation utilized in experimental tests on ground^57^. Doses expected from the background radiation are not as high as what may occur during Solar Particle Events (SPEs). In these cases, analysis of the likely exposure levels for skin and blood forming organs with expected shielding levels are likely to remain significantly below 1 Gy-Eq^78^. Because of this NASA’s Acute Radiation Risk which was already green (assessed as low) at all levels was merged into the In-Flight Medical Risk in 2020, however, radiation does contribute to long term health effects such as cataracts in this population^2,3,78^.

There is little published data on limiting the effect of heavy ion (HZE) particle effects on eye tissue, however, there is evidence of the serious effects of different levels of radiation on ocular tissues^71,79^. Studies have demonstrated radiation induced destruction of aromatic and sulfur-containing amino acids, aggregation, crosslinking, dissociation, fragmentation, and partial folding with an effect on ocular tissue^80^. In particular, crystallins, the main protein constituent of lenses, are impacted by pronounced aggregation of protein and destruction of aromatic amino acid residues with a dose-dependent decrease of fluorescence intensity altering the shape of aromatics^80^. This in turn affects the transparency of the lens and increases occurrence of cataracts^80^. While cataractogenesis is the most frequently observed finding secondary to radiation exposure, corneal epithelial damage, conjunctival erythema, conjunctivitis, keratitis, corneal ulceration, iritis, and retinal edema have all been found after acute radiation exposure^64,81^. Radiation exposure has also been demonstrated to have a profound affect on the optic nerve, with a robust literature demonstrating increased risk for optic neuropathy after external beam radiation therapy. Through such studies, the maximum radiation dose to the anterior visual pathway has been established as a significant determinant for the development of radiation-induced optic neuropathy, and the anterior visual pathway has been shown to tolerate no >50 Gy of cumulative radiations in fractions <2 Gy^82–89^. Furthermore, even in the absence of acute radiation damage in space, it is unclear how such exposures will affect ocular tissue long term with prior studies investigating the long-term effects of radiation therapy to the eye demonstrating tissue necrosis, decreased tear production, scleral melting, cataracts, corneal neovascularization, posterior segment neovascularization, radiation retinopathy, and radiation-induced cancers^81^. Updated NASA Standards limit exposures for American crew to 600 mSv over a career while other International Space Agencies allow up to 1000 mSv^90^. Most ISS missions are well below this amount, but a 1–2 year Mars mission is likely to exceed this limit^2^. Therefore, future studies characterizing the true effect of the galactic cosmic radiation on ocular tissues are essential, with a specific focus on preventative measures.

#### Diagnosis and treatment

Diagnosis typically depends on a combination of clinical exam with slit lamp and dilated fundus exam, symptoms, and history of radiation exposure. Treatment is classically reactive, such as cataract surgery for radiation-induced cataracts, lubrication for radiation induced dry eye. However, radiation-induced changes may also not be salvageable in the case of retinal or optic nerve damage^91^. Astronauts who experience these Long Term Health effects are likely to do so post-flight and post-career where they will have access to terrestrial interventions. These considerations receive lower prioritization for in-flight CHP systems.

### Non-ionizing radiation (laser and/or sunlight eye injury)

#### Definition

Laser and sunlight irradiation of the eye may cause damage to the cornea, lens, or retina depending on the wavelength of light and differential energy absorption of the ocular tissues.

#### Potential causes of injury and known data on injury in spaceflight

Non-ionizing radiation includes laser and sunlight injury that can occur through windows or a helmet. While laser eye injury may not classically occur secondary to exposure of Astronauts in space, there is potential for laser eye injury to occur due to space related activities. The use of lidars, a laser radar for light detection and ranging, is considered in a number of missions conducted by NASA. Lidars serve a variety of uses including measuring a range of atmospheric or earth surface properties^92^. Part of the laser radiation may be scattered or absorbed by the atmosphere with the remaining laser radiation emitted from the spacecraft directed at earth. The risk for ocular injury to an exposed eye is quite low, however, exposure to the eye via a large telescope may result in ocular damage to the cornea, lens, and/or retina^64^. Military laser usage may serve as a correlate of this laser exposure with evidence of the damaging effects of “unknown strong light” on the retina, however, the extent of injury is very dependent on the specific wavelength parameters and include visible and near infrared lasers^93,94^. Sunlight concerns are addressed by special coatings on visors of Extravehicular Mobility Units (EMUs) and windows on vehicles to prevent ocular injury.

#### Diagnosis and treatment

Early Diagnosis of laser eye injury is challenging as initial presentation of cases may be notable for the lack of significant abnormalities on fluorescein angiography^95^. However, Optical Coherence Tomography (OCT) may delineate the size and extent of retinal involvement from exposure to laser eye injury. In these cases, prompt referral of these cases is necessary and classically involves a rapid initiation of medical therapy, including 10–14 day combined courses of steroid and non-steroid anti-inflammatory medication to enhance the initial recovery of vision and reduce the likelihood of longer term complications from scarring and neovascularization^95^.

### Diagnostic modalities

Pre- and post-flight testing of ocular function is extensive, particularly as it relates to prevention and monitoring of spaceflight associated neuro-ocular syndrome^57^. When considering both SANS, and the ocular traumas and emergencies detailed above, the diagnostic modalities *during* spaceflight are of the utmost importance. Existing Spaceflight Data Abilities during mission include vision questionnaires, Visual Acuity testing, Amsler Grid testing, Fundoscopy and Fundus Photography, Orbital Ultrasound, and Optical Coherence Tomography (OCT)^57^.

### In flight vision questionnaire

During space flight, each crew member completes an in-flight vision questionnaire including questions about distortion, vision in dim light, fluctuation in visual acuity, depth perception, double vision, transient vision loss, and change in near, intermediate and distance vision, as well as questions regarding type of eyewear used and symptoms of headaches, tinnitus, nausea or impairment in cognition^57^. For each of these, crew members are asked to rate change as mild (does not affect daily activities), moderate (crewmember had to make changes to accommodate completion of activities), or severe (changes have significantly affected or interfered with completion of daily activities)^57^.

### Visual acuity testing and Amsler grid testing

During space flight, far visual acuity is tested for each eye with and without a corrective lens using a software application loaded onto the OCT laptop which provides a screenshot of a standard logMAR Snellen chart^57^. Testing involves positioning the astronaut 15 feet away from the OCT laptop. Ground support personnel are able to control the software remotely by accessing the OCT laptop. A similar software and procedure are executed for Amsler grid testing to assess for macular degeneration or edema (any changes to the appearance of the grid such as wavy, blurred, or missing lines are noted to be abnormal). Near visual acuity is also tested for each eye with and without corrective lenses. This is executed by placing a paper-based eye chart 16 inches from the astronaut on the wall of the laboratory module. Refractive error may also be assessed in-flight to gauge appropriate glasses prescription as refractive error may change in-flight. Apart from their role in assessing SANS-related progression, visual acuity testing and Amsler grid testing are helpful in assessing any ocular complaint from corneal abrasion to lens dislocation to retinal pathology.

### Fundoscopy and fundus photography

Dilated fundoscopy is performed on each eye to obtain images of the retinal surface and optic disc. These in-flight exams are remotely guided using a fundoscope and desktop streaming software technology with still images and short video clips recorded and downlinked^57^. Fundus photography has been particularly helpful in assessing the posterior segment findings of choroidal folds, optic disc edema and pallor, cotton-wool spots, and disc edema in the monitoring, prevention and treatment of SANS^57^.

### Orbital ultrasound

During flight, ocular ultrasound is conducted on days 30, 90, and 30 days before returning to earth. These in-flight sessions are guided remotely by sonographers in mission control. Utilizing real-time cabin video and ultrasound feeds from ISS, sonographers are able to guide astronauts onboard to acquire the images on one another with expert guidance^57^. The application of ultrasound in ophthalmology are vast including biometry to measure intraocular distances, bio-microscopy with ultra-high-frequency probes, and general scanning of the anterior and posterior chamber in the eye^96–98^. Furthermore, the unique constraints of the space environment necessitate novel diagnostic and therapeutic techniques, which ocular ultrasound may be particularly suited for^99^. The ultrasound examination could be used to exclude ocular pathology such as globe disruption, lens dislocation, ocular foreign body, retinal/choroidal detachment, or occlusion of the retinal artery, and has been demonstrated to be effective in quantifying the pupillary light reflex with B- and M- mode ultrasonography, which could be used to gauge pupillary reaction times in patients with central nervous system injuries or alterations in the autonomic nervous system^96^.

Crew members may complete a comprehensive ocular examination using B- and M-mode ultrasonography with remote guidance from experts in mission control, obtaining multiple anteroposterior, oblique, and coronal views of the eye^96^. However, this was dependent on a satellite transmission delay for both video and audio of only a couple of seconds. In cases of acute trauma to the eye, acute ocular foreign body, abrasion, irritation, or vision loss during periods of time when communication with mission control is asynchronous, effective utilization of this modality may likely be more challenging without the active expert guidance. This also applies in interplanetary travel to Mars for example when communication delays are unavoidable^7^. In addition, ultrasonographic assessment is limited in its ability to assess ocular surface injury. For example, while it may allow for assessment of lens position, heme, and lacerations, it is limited in its ability to assess the surface of the eye, and more specifically, the cornea/anterior segment of the eye. In addition, as mentioned above, if full thickness laceration or globe injury is suspected, ultrasonographic assessment would be contraindicated for fear of iatrogenic extrusion of ocular contents. Therefore, while it may assess anatomic positioning, pupillary reaction to light, lens placement, and large vitreous and retinal abnormalities, it may not be sufficient to assess the most common ocular complaints of foreign body, abrasion, irritation, tearing, etc (Table 1).

### Optical coherence tomography

OCT is a diagnostic imaging modality used to measure the retinal thickness, volume, and retinal nerve fiber layer (RNFL) thickness by quantitative cross-sectional analysis. OCT is conducted before, during, and after space flight to detect changes primarily in the posterior segment of the eye, including the optic disc, RNFL, retinal pigment epithelium (RPE), and the vascular choroid. In-flight, the OCT scans are performed with subjects placing their chin on a chin rest, while the device performs a scan of the eyes with ground-support personnel able to remotely access the OCT laptop^57^.

OCT is most commonly used in space flight to evaluate changes associated with visual impairment, syndromes of elevated intracranial pressure, and SANS. Although OCT is not the first line modality to evaluate anterior segment structures terrestrially, with specific instruction, it may be applied in space to view the anterior segment^100,101^. With a protocoled method of expert guidance of novice users, with step by step instructions by optometrists as subject matter experts, flight surgeons were able to execute anterior segment OCT module imaging of the cornea, sclera, and anterior angle/anterior chamber. Anterior eye pathologies evaluated included corneal abrasions and ulcers, scleritis, and acute angle closure glaucoma^100^. These findings are promising, leveraging the versatility of OCT and both improving diagnostic capabilities of the more common anterior segment complaints on ISS and limiting excess equipment on the vehicle. Similarly, a case series examining 38 eyes with different types of ocular injuries (penetrating injury, perforating injury, intraocular foreign body, ocular burn, contusion, and lamellar laceration) found that even in situations where slit-lamp examination did not provide a clear diagnosis, OCT was able to^101^. Particularly in settings of opaque corneas (secondary to contusion/trauma/blood), OCT proved useful, capable of assessing acute angle closure with pupillary block, blood infiltrating the cornea, descemet membrane corneal abnormalities, corneal edema, corneal abrasions, localization and extent of penetration of foreign bodies, etc^101^. As with ultrasound on ISS, one concern is that in the absence of remote synchronous guidance during segments of space flight, this tool may not prove as helpful in assessing acute time-sensitive ocular traumas, necessitating automated AI-based techniques or inflight training modalities to guide OCT image capture and diagnosis.

### Future considerations on diagnostic modalities

As discussed, many of these in-flight tools suffer from inadequate assessment of the surface of the eye, and dependence on remote synchronous image capture and diagnosis guidance from experts in mission control. When considering addition to the diagnostic modalities, it is also important to note the many constraints placed on equipment used in space flight, namely considerations of radiation resistance, ability to function as a closed-loop system, requirement for low weight/volume, function in microgravity, and inter- and intra-user variability.

### Anterior surface evaluation with fluorescein examination

Complaints related to the anterior surface of the eye (eye tearing, irritation, debris, dryness) are some of the most common among astronauts during space flight (Table 1). However, the modalities present in current space flight (OCT and ultrasound) do not have the capacity to thoroughly assess ocular surface abnormalities that may be contributing to these symptoms. Fluorescein strips/dye may be utilized to assess any corneal or conjunctival irregularities, dryness (tear break up time), foreign bodies, and even globe injuries (seidel test)^31^. It is helpful in both identifying and monitoring corneal epithelial defects, corneal ulcers, and corneal abrasions. It is advantageous in that it comes in ready to use ophthalmic strips with incorporated dye and does not cause ocular irritation when used topically. Simple Pen light examination may also reveal macroscopic changes in the eye as a screening exam. Inflammation, irritation, and venous congestion may cause discharge, hyperemia of the conjunctiva and swelling of the conjunctiva and lids on visible on pen light exam. Dryness will demonstrate loss of corneal and conjunctival luster on pen light exam and trauma may preset with a visible hemorrhage. However, anterior segment examination tools such as a portable slit lamp or anterior segment imaging attachment are still highly valuable.

### Handheld portable slit lamp examination and photography

The handheld portable slit lamp exam is commonly used in consultations throughout the hospital, particularly suitable for bedside, home, or clinic exam where the large slit lamp may not be readily available^102^. It provides reliable diagnostic images for anterior segment disease of the conjunctiva, cornea, and lens^102–106^. However, more subtle corneal, conjunctival, and lid abnormalities may still not be identified well^106^. When considering the most common ocular complaints, namely foreign body, ocular irritation, corneal abrasion, the portable slit lamp may prove particularly useful as it allows for rapid inspection of minute foreign bodies and the surface of the eye to assess for small particles or irregularities that may be contributing to symptoms^102^. It is also small and lightweight (e.g. ~220(W) × 95(D) × 220(H) mm, ~750 g versus 312(W) x 305(L) x 676(H) mm, ~12 kg), and ergonomically ideal addition to the current on-flight diagnostic modalities. In addition, camera attachments could allow for communication with mission control to facilitate diagnosis^106^. However, it does require significant training, and as is, the portable slit lamp does not allow for easy synchronous guidance from mission control to facilitate a thorough examination.

### Anterior segment camera—handheld devices

There are multiple remote handheld imaging devices on the horizon for use on earth with potential extension to the space environment^107^. Multiple companies including Paxos Scope, D-Eye, Tesseract Health, Zeiss, Eyefficient, Volk, Nidek, Topcon and more have been developing hand-held retinal imaging devices with capacities to obtain images of the fundus, retina, with some even developing methods for improved vascular imaging^108^. These lightweight, handheld retinal or fundus cameras carry an added time-saving benefit of eliminating the 20 min of patient dilation time needed. They also allow for efficient image capture and sharing, which would support communication with mission control. One key feature supporting extension of these tools into space is the built in image quality analysis to ensure quality photos without the need for synchronous feedback from mission control. These systems leverage machine learning and artificial intelligence (AI) based technologies to allow for automated image processing and analysis which would enable closed loop in-flight monitoring^109^. Even smartphone ophthalmic imaging has become increasingly popular, particularly in less-advantaged regions due to the easy portability, ubiquity, low cost, and diagnostic efficacy compared D-Eye, Peek Retina, Paxos Scope, Fundus on phone, and iExaminer have also developed smartphone ophthalmic imaging attachments which are able to obtain non-mydriatic fundus images and offer 6 degree fields of view in undilated pupils, capable of delineating the optic nerve and posterior pole^108^. These attachments benefit from portability, low cost, and diagnostic efficacy, and allow for easy transmission of images and AI-based detection of certain biomarkers^110^. However, these imaging techniques and platforms require further validation in humans before we can be confident in their ability to assess both anterior and posterior segment abnormalities in mission.

### Tear assessment

In the dry eye literature, assessment of the tear film is routinely utilized with non-invasive, reliable measures^111–116^. In particular, when considering dry eye as a potential contributor to ocular irritation in space, modalities such as the Schirmer test, noninvasive tear break up time, and tear film oriented diagnosis, fluorescein break up pattern may be of use. The Schirmer test involves placing a piece of filter paper at the lateral 1/3rd of the lower fornix and assessing the level of wetting of the filter paper after 5 min^117^. Noninvasive tear break up time involves placing one drop of fluorescein dye in the lower fornix and asking the patient to blink multiple times, after which the cornea is examined under blue light on slit lamp exam for dry spots^118^. Tear film oriented diagnosis and fluorescein break up pattern help differentiate between aqueous deficiency, decreased wettability, and increased evaporation as causes for dry eye symptoms and irritation^115^. Newer diagnostic modalities include hand held devices such as ipen and tear lab osmometers, which assess the contribution of osmolarity to dry eye irritation. Considering such tear assessments such as the Schirmer test and noninvasive tear break up time, which require barely any addition of equipment on ISS, may help better diagnose and manage the underlying cause of ocular irritation astronauts are experiencing as a function of their environment. However, it is always integral to consider any additional mass in the context of spaceflight clinical need, and symptomatic treatment on ISS with artificial tears and ointments may not be substantially altered by additional information provided by these technologies.

### Artificial intelligence

In both the space literature and ophthalmology literature, artificial intelligence (AI), and more specifically machine learning using artificial neural network structure may allow for improved asynchronous diagnostics as technology and algorithms improve. In particular, AI has been studied to support therapy decision-making in age-related macular degeneration by training machine learning algorithms using OCT imaging and analyzing different features of the scan^119^. Similarly, AI has demonstrated promise in its ability to detect sub-clinical features of corneal ectasia, clinical decision making in glaucoma, and diagnosing diabetic retinopathy^119,120^. AI capabilities have similarly extended to periorbital diagnosis and treatment, with studies on the horizon investigating the ability of trained algorithms to detect periocular cancers and lid malposition. Given the limitations of synchronous care in exploration space flight, AI abilities may expand the diagnostic capacity of astronauts, leading to improved recognition and treatment of ocular injuries and exposures^121,122^.

### Therapeutic modalities

#### Current treatment modalities

In addition to the diagnostics outlined above, current treatment modalities on ISS include pupil dilating (e.g. phenylephrine) and numbing (e.g. proparacaine) eye drops, artificial tears (refresh), magnifying glasses, glasses and contact lenses^29,123^. Of note, proparacaine must be refrigerated, which limits the shelf life of the medication in exploration missions which have challenges with up mass and inability to keep certain medications refrigerated.

When examining for corneal abrasion or foreign body, 1–2 drops of proparacaine ophthalmic solution will be inserted into the eye. 1–2 drops of artificial tears will then be placed on a fluorescein strip, which is then applied to the lower, inner eyelid until yellow-green film covers the eye. The Blue light setting on the ophthalmoscope will then be applied and areas of more intense staining are a sign of an epithelial defect, suggestive of issues such as dryness, foreign body, abrasion or laceration. In cases of abrasion, artificial tears are utilized to rinse the eye. Flight surgeons (non-ophthalmologists) are contacted to provide support from the mission control center. If a foreign body is visualized, attempts to remove it are done by flushing the eye with artificial tears^29,123^. If unsuccessful, a moistened cotton swab is used to attempt to gently dislodge the foreign body by rotating the cotton swab. Ciprofloxacin ophthalmic solution is utilized for topical ophthalmic use (1 drop in the affected eye 4 times daily) when needed for issues such as abrasion. Cyclogyl (cyclopentolate) ophthalmic solution may also be used to decrease pain and light sensitivity through pupillary dilation. If signs of infection are present, eye patching is contraindicated, however, a shield or eye pad over the affected eye may be placed for protection^123^. If there is a penetrating eye injury, astronauts are advised to neither remove the object nor any tissue extruding from the eyeball. A single eye pad is placed lightly over the injured eye with eyelid closure and a metal eye shield is taped over the eye pad without putting pressure on the eye, followed by closing and patching the unaffected eye with the eye pad^123^. A flight surgeon is then contacted and the astronaut would likely need to be evacuated.

In cases of eye infection, the white of the eye is observed for redness, and astronauts are assessed for blurred vision, pain, abnormal sensitivity to light, and a hypopyon (accumulation of white blood cells and fibrin) in the eye. At this point, a flight surgeon is typically contacted, however, it is important to note that this flight surgeon is not an ophthalmologist and typically provides support from the mission control center. Antibiotic choices on ISS include polytrim ophthalmic solution and ciprofloxacin ophthalmic solution. Polytrim ophthalmic solution is considered to be first line on ISS, with a dose of 1 drop on the affected eye 6 times daily^123^. If there is no improvement in eye examination or treatment, ciprofloxacin ophthalmic solution is applied 1 drop in the affected eye 4 times daily^123^. In cases of herpetic eye infection (branch-like pattern on fluorescein examination), VIRA-A (vidarabine ophthalmic ointment) is applied instead of antibacterial ointment 5 times a day at 3 h intervals^123^. Contact lenses are avoided until complete resolution of symptoms and examination. Of note, vidarabine does cause ocular surface toxicity, and therefore extended use of vidarabine may exacerbate any underlying surface irregularity or irritation. Acyclovir ointment may also be utilized in these cases, with less risk for ocular surface toxicity.

Further examination of the cornea for corneal ulcer would involve a fluorescein examination. In these cases, eye patching is avoided and ciprofloxacin ophthalmic solution use is advised, 2 drops every 15 min for 6 h, then 2 drops every 30 min, followed by 2 drops every hour the second day and 2 drops every 4 h the third day until all medication is used^123^. Of note, when considering drop application, it is important to take into account the amount of drop the eye can feasibly absorb. Although 2 drops at a time have been prescribed in spaceflight, on earth, placing more than one drop at a time would rarely be recommended, as the eye cannot actually hold more than one drop.

In the case of chemical burns, drink bag containers full of water, drink straws, towels, eyewash, tubing connectors, wastewater collection bags, and proparacaine ophthalmic solution (eye drop) are utilized. First eye irrigation is attempted with the drink bag and drink straw, deploying the eyewash through tubing containers for definitive eye flush, and anesthesia of the eye only if needed with 1–2 drops of proparacaine^123^. Goggles with continuous flush are then applied with a wastewater collection bag and surgeons are contacted in ground control. After flushing, fluorescein examination is performed with cyclopentolate eye drops for pupil dilation and ciprofloxacin ophthalmic solution is instilled into affected eye 4 times daily^123^. Ibuprofen, acetaminophen, and hydrocodone/acetaminophen are also available on ISS for additional analgesia. Of note, on earth in cases of chemical injury, the first step classically involves utilizing a pH strip to gauge the pH of the surface of the eye. If the pH is not 7, irrigation will be performed with the goal of returning pH to 7. This process can involve as much as 8-10 L of fluid of irrigation. Due to fluid availability restriction and microgravity, this process may get more complicated on ISS; however, including assessment of pH may facilitate a pH goal-directed irrigation process and improve the ocular outcome.

In exploration spaceflight where resupply is compromised, communication delays present, and evacuation either delayed or absent, these approaches used in the LEO paradigm may become inadequate^7^. Additionally, the issue of medication stability can become significant given storage requirements and duration of missions^78,124^. As needs for crew autonomy increase with distance from Earth, strategies must be developed to ensure that the acute response to ocular medical issues will be as robust as possible.

### Protective/preventative measures

Given the potential for considerable short-term symptoms and long-term effects of ocular injury in space, preventative measures have been the cornerstone of medical care for astronauts to date.

### Protective eyewear (Goggles)

The emergency eyewash system design includes a pair of swim goggles and tubing to allow for high flow water eyewash delivery. During EVAs, crew members wear two-piece semi-rigid spacesuits. During cargo loading, EVA suit changes, and maintenance activities during spaceflight, eye protection could be worn more frequently to further protect from foreign body abrasion or particulate (e.g. lunar dust) induced ocular irritation. Goggles would ideally be lightweight, flexible and slip-free for comfort and lack of irritating pressure points, however, although provided, they are not routinely worn by astronauts^125^. It is also important for any eyewear protection to be devoid of hinge screws which could become dislodged during an EVA leading to choking, oral/nasal ingestion, or even mechanical suit failure^125^. Ideally, the lens would offer 360 degree protection without risk of particles getting trapped under the lens, as well as a soft-brown tint and bi-layer mirror to improve color perception and protection against harmful infrared light exposure^125^.

### Fish oil—omega 3

Fish oil supplementation has been investigated as an adjunctive treatment for dry eye disease, and may also be helpful to mitigate the microgravity induced changes to the ocular surface that may predispose to irritation and epithelial disruption. Omega-3 fatty acid supplements are safe with relatively few side effects in standard dose^126^. Across research studies, the doses classically include 180 milligrams of eicosapentaenoic acid and 120 mg of docosahexaenoic acid twice daily. High doses may be associated with increased bleeding risk, higher levels of low-density lipoprotein, and irregular blood glucose control, which may be problematic during long-duration spaceflight.

### Corneal shield/protective contacts

Collagen corneal shields are utilized to protect the ocular surface after surgery or in traumatic and non-traumatic corneal conditions. Studies have suggested that collagen shields may be employed to enhance drug delivery and corneal wound healing^127^. Similarly, patients with acute nonsurgical epithelial problems, such as contact lens abrasions and recurrent erosion have responded well to the use of the collagen shield with improved healing^128^. However, patients with chronic epithelial defects have responded poorly, likely secondary to underlying abnormalities in one of the layers of the cornea preventing epithelial growth in the area of the defect^128^. No infections were noted in association with the placement of corneal shields. Protective contacts may also be considered for additional protection during relatively riskier EVA or in-flight activities in which helmets may inhibit astronaut ability to protect their eyes from small particles. However, long-term contact lens use may carry an additional risk of infection or epithelial, stromal, or endothelial compromise^129^.

### Preservative Free Artificial Tears (PFAT)

Artificial tears may be used on flight in cases of corneal abrasion, foreign body sensation, irritation, dryness, or dust exposure. However, artificial tears are not used daily or in a preventative fashion^123^. It is possible that in spaceflight, astronauts may experience altered sensation or dryness, with altered ocular surface and blinking in microgravity. This in turn may lead to increased susceptibility to dryness and epithelial disruption in response to air particulates. Apollo astronauts recommended that saline eye drops be provided in large quantities noting that “…ocular irritation from the lunar dust required a lot of saline irrigation to treat”^130^. Preservative free artificial tears—both carboxymethylcellulose and hyaluronic acid artificial tear formulations—could be used more frequently 4-6 times a day to keep the ocular surface lubricated, supplement deficient tear film, and improve ocular comfort and optimize visual function^131^.

### Routine exams

As discussed, there is ample opportunity for small particles to get into the eye or become lodged under the eyelids causing recurrent subclinical irritation or abrasion. In this case, routine examination with fluorescein dye to assess for surface irregularity may be helpful in order to diagnose and treat abrasions with lubrication, antibiotic treatment, and/or foreign body removal as needed earlier to decrease irritation time and improve outcomes.

### Outlook and summary

This review characterizes ocular injury, detection, and management in space flight, as well as future directions based on current innovations in the field of ophthalmology. Ocular conditions, injuries and subsequent ocular discomfort can cause serious discomfort to astronauts and it is integral to better report ocular symptoms and diagnoses during space-flight and treatment. This study provides a review of publicly available data and literature surrounding ocular conditions and injuries in space, however, it was limited in that the authors did not have access to further clinical information surrounding reported complaints, or reported diagnostics and management, as well as associated limitations, for these cases. Future studies would benefit from exploration of diagnostic modalities and limitations of available testing as they pertain to specific actual injuries experienced in space flight, as well as from a more in depth report of the clinical cases.

Given the unique nature of human spaceflight, there are no available estimated deadlines for restoring visual performance after treatment of eye injuries. These are all considered on a case-by-case basis. Expedient treatment of ocular injury in Spaceflight is essential to decrease performance impairment in the short-term and avoid long-term visual impairment. Hindrances to visual performance pose immediate increased risk to the crew and mission. Inflight eye injury could also lead to permanent debilitating visual impairment. On the ISS, in-mission eye injuries are supported telemedically from mission control by flight surgeons with specialist support as needed. Clinical guidelines within NASA are not publicly available for review. Post flight, ocular changes due to SANS are considered when recertifying astronauts for subsequent flights, although the authors are not aware of any cases of eye injury that have affected astronaut certification to date outside of SANS.

The shift from earth-tethered to earth-independent medical management for human spaceflight is dictated by limited evacuation capabilities as well as considerations of increased risk. Upcoming Lunar (and future Deep Space) missions pose multiple challenges. Medical evacuation, currently difficult from LEO, is an even more challenging feat on Lunar Missions. Lunar missions can evacuate, but the timeframe for that evacuation can range from 3–11 days. Therefore, without supporting capability designed into the system, there is a high likely hood of poor outcome for time sensitive ocular injuries. Increased risk of ocular injury on these missions is anticipated due to: longer duration in spaceflight environment; increased frequency of transitions between habitats; and greater exposure to planetary surfaces. In light of expected increased injury risk and decreased evacuation capacity, immediate onboard treatment capabilities are essential to avoid severe consequences from ocular trauma.

Ocular issues in exploration spaceflight back to the moon and on to Mars will pose unique challenges because of stricter mass and volume allocations for medical capabilities, loss of real-time communication, and delayed or absent opportunities for evacuation of injured astronauts. Consideration of a broad set of ocular injuries and conditions enables a more thorough assessment of appropriate technologies for inclusion in a CHP system depending on the needs of a given mission. It is important to note that it may not be just resources that limit the ability to diagnose and treat in space, but also knowledge, skills and abilities as it is not guaranteed that any physician, much less an ophthalmologist, will be present in missions where we need to consider whether certain treatments would be effective or may cause more harm than good. Through this review, we hope to encourage a holistic team approach between NASA and the ophthalmology community to improve ocular health in space flight, and to encourage greater awareness and public report of data related to ocular symptomatology.

## Data Availability

All data derived from NASA evidence reports and publications reviewed are publically available.
